# Mitochondrial and autophagic alterations in skin fibroblasts from Parkinson disease patients with Parkin mutations

**DOI:** 10.18632/aging.102014

**Published:** 2019-06-09

**Authors:** Ingrid González-Casacuberta, Diana-Luz Juárez-Flores, Mario Ezquerra, Raquel Fucho, Marc Catalán-García, Mariona Guitart-Mampel, Ester Tobías, Carmen García-Ruiz, José Carlos Fernández-Checa, Eduard Tolosa, María-José Martí, Josep Maria Grau, Rubén Fernández-Santiago, Francesc Cardellach, Constanza Morén, Glòria Garrabou

**Affiliations:** 1 Laboratory of Muscle Research and Mitochondrial Function, Institut d’Investigacions Biomèdiques August Pi i Sunyer (IDIBAPS), University of Barcelona (UB), Department of Internal Medicine, Hospital Clínic of Barcelona (HCB), Barcelona 08036, Spain; 2 Centro de Investigación Biomédica en Red (CIBER) de Enfermedades Raras (CIBERER), Madrid 28029, Spain; 3 Laboratory of Neurodegenerative Disorders, IDIBAPS, UB, Department of Neurology, HCB, Barcelona 08036, Spain; 4 CIBER de Enfermedades Neurodegenerativas (CIBERNED), Madrid 28031, Spain; 5 Cell Death and Proliferation, IDIBAPS, Consejo Superior Investigaciones Científicas (CSIC), Barcelona, Spain; 6 Liver Unit, HCB, IDIBAPS and CIBER de Enfermedades Hepáticas y Digestivas (CIBEREHD), Barcelona, Spain; 7 USC Research Center for ALPD, Keck School of Medicine, Los Angeles, CA 90033, USA

**Keywords:** Parkinson’s disease, Parkin mutation, mitochondrial function, autophagy, fibroblasts

## Abstract

PRKN encodes an E3-ubiquitin-ligase involved in multiple cell processes including mitochondrial homeostasis and autophagy. Previous studies reported alterations of mitochondrial function in fibroblasts from patients with PRKN mutation-associated Parkinson’s disease (PRKN-PD) but have been only conducted in glycolytic conditions, potentially masking mitochondrial alterations. Additionally, autophagy flux studies in this cell model are missing.

We analyzed mitochondrial function and autophagy in PRKN-PD skin-fibroblasts (n=7) and controls (n=13) in standard (glucose) and mitochondrial-challenging (galactose) conditions.

In glucose, PRKN-PD fibroblasts showed preserved mitochondrial bioenergetics with trends to abnormally enhanced mitochondrial respiration that, accompanied by decreased CI, may account for the increased oxidative stress. In galactose, PRKN-PD fibroblasts exhibited decreased basal/maximal respiration vs. controls and reduced mitochondrial CIV and oxidative stress compared to glucose, suggesting an inefficient mitochondrial oxidative capacity to meet an extra metabolic requirement. PRKN-PD fibroblasts presented decreased autophagic flux with reduction of autophagy substrate and autophagosome synthesis in both conditions.

The alterations exhibited under neuron-like oxidative environment (galactose), may be relevant to the disease pathogenesis potentially explaining the increased susceptibility of dopaminergic neurons to undergo degeneration. Abnormal PRKN-PD phenotype supports the usefulness of fibroblasts to model disease and the view of PD as a systemic disease where molecular alterations are present in peripheral tissues.

## Introduction

Parkinson’s disease (PD) has become increasingly prevalent as the population ages, being the second most prevalent neurodegenerative disorder worldwide [1]. Homozygous and compound heterozygous mutations in the Parkin gene (*PRKN*) are the most common cause of recessively inherited early-onset PD, accounting for up to 50% of familial PD and about 15% of sporadic PD (sPD) with disease onset before 45 years [2]. Parkin-associated PD (PRKN-PD) is clinically similar to sPD besides some specific clinical features and the significant earlier age of onset, which can occur from childhood to the fourth or fifth decade of life [3]. The neuropathological hallmark of PRKN-PD is as in sPD, the prominent death of dopaminergic neurons (DAn) in the *substantia nigra pars compacta* (SNpc). In contrast, the presence of Lewy bodies in PRKN-PD is infrequent [3–5]. The etiopathogenesis of PD has been associated to several molecular events including mitochondrial dysfunction and autophagy impairment [6,7], which may compromise neuronal survival.

The Parkin protein (PRKN) is a multifunctional E3 ubiquitin ligase that exerts crucial neuroprotective functions in DAn [8,9]. A key role of PRKN in mitochondrial macroautophagy (mitophagy) has been reported in different models of the disease [10–14]. Specifically, upon mitochondrial depolarization there is a reduced turnover of the PTEN induced putative kinase 1 protein (PINK1) and thus, it accumulates in the outer mitochondrial membrane leading to the recruitment and phosphorylation of PRKN. Subsequently, PRKN mediates the polyubiquitination of many outer mitochondrial membrane proteins. Briefly, the polyubiquitination is the signal for the recruitment of adaptor proteins such as p62, which allows the binding of the microtubule-associated protein 1 light chain 3 (LC3BII) in the forming autophagosome to initiate mitochondrion sequestration and the following clearance upon fusion with the lysosome [7,15]. Recent evidence suggests that PRKN is also involved in the aggresome-macroautophagy pathway in which it promotes the sequestration of misfolded proteins into aggresomes and its consequent clearance by autophagy through p62 and LC3BII recruitment [16,17]. In summary, growing evidences point out PRKN as a crucial player in the different pathways that consitute macroautophagy (hereafter called autophagy).

In this scenario, a number of studies have postulated that PRKN-PD may derive from the impaired clearance of bioenergetically compromised mitochondria [8,18] and the consequent accumulation of dysfunctional mitochondria that trigger an overproduction of intracellular reactive oxygen species (ROS) that eventually may harm cell components. Concurrently, other PRKN protein substrates may accumulate [8,19] within the cells, eventually compromising their viability [6,20]. Yet, the precise mechanisms by which *PRKN* loss-of-function mutations lead to neurodegeneration remain elusive.

A major challenge to study PD is the inaccessible nature of the specific neural cell types targeted by the disease which are only available only *post-mortem*. In this context, the development of validated models to study PD is crucial to enable the understanding of the molecular pathways underlying the disease pathogenesis. On the other hand, there is increasing evidence that PD pathology is not confined only in the central nervous system (CNS) but is also present in the peripheral autonomous nervous system and the organs that the latter innervates, including the skin [21]. Skin-derived fibroblasts are accessible peripheral cells that constitute a patient-specific cellular system that retain the genetic background of the patients and potentially preserve the environmental and cumulative age-related events in addition to show relevant expression of most PARK genes [22,23]. Moreover, they can potentially recapitulate some pathophysiological features of the disease^22^. In fact, many of the molecular hallmarks occurring in PD-DAn have been reported in fibroblasts from patients with sporadic and monogenic forms of the disease [24–27]. Notwithstanding, previous studies on mitochondrial function in PRKN-PD fibroblasts have been only conducted in glycolytic conditions in which fibroblasts generate most of cell ATP via anaerobic glycolysis, thus potentially masking mitochondrial alterations present in these cells [28]. In case of PRKN-PD fibroblasts phenotyping, these studies have reported controversial outcomes without assessing, in parallel, the cellular impact of *PRKN* mutations on autophagic flux [29–34]. Therefore, growing PRKN-PD fibroblasts in a glucose-free medium such as galactose, which has been previously used for the diagnosis of primary mitochondrial diseases [35,36], may provide a closer approach to the more oxidative metabolism and the potential associated mitochondrial function alterations present in the DAn of PRKN-PD patients.

In the present study, we aimed to characterize mitochondrial function and autophagy in skin-derived fibroblasts from PRKN-PD patients in parallel in glycolytic (glucose) and mitochondrial-challenging conditions (galactose). The identification of alterations in PRKN-PD fibroblasts under mitochondrial-challenging conditions may provide insight into disease pathogenesis, as the specific neural cell types targeted by the disease are predominantly oxidative.

## RESULTS

### Mitochondrial respiration

**Figure 1:**
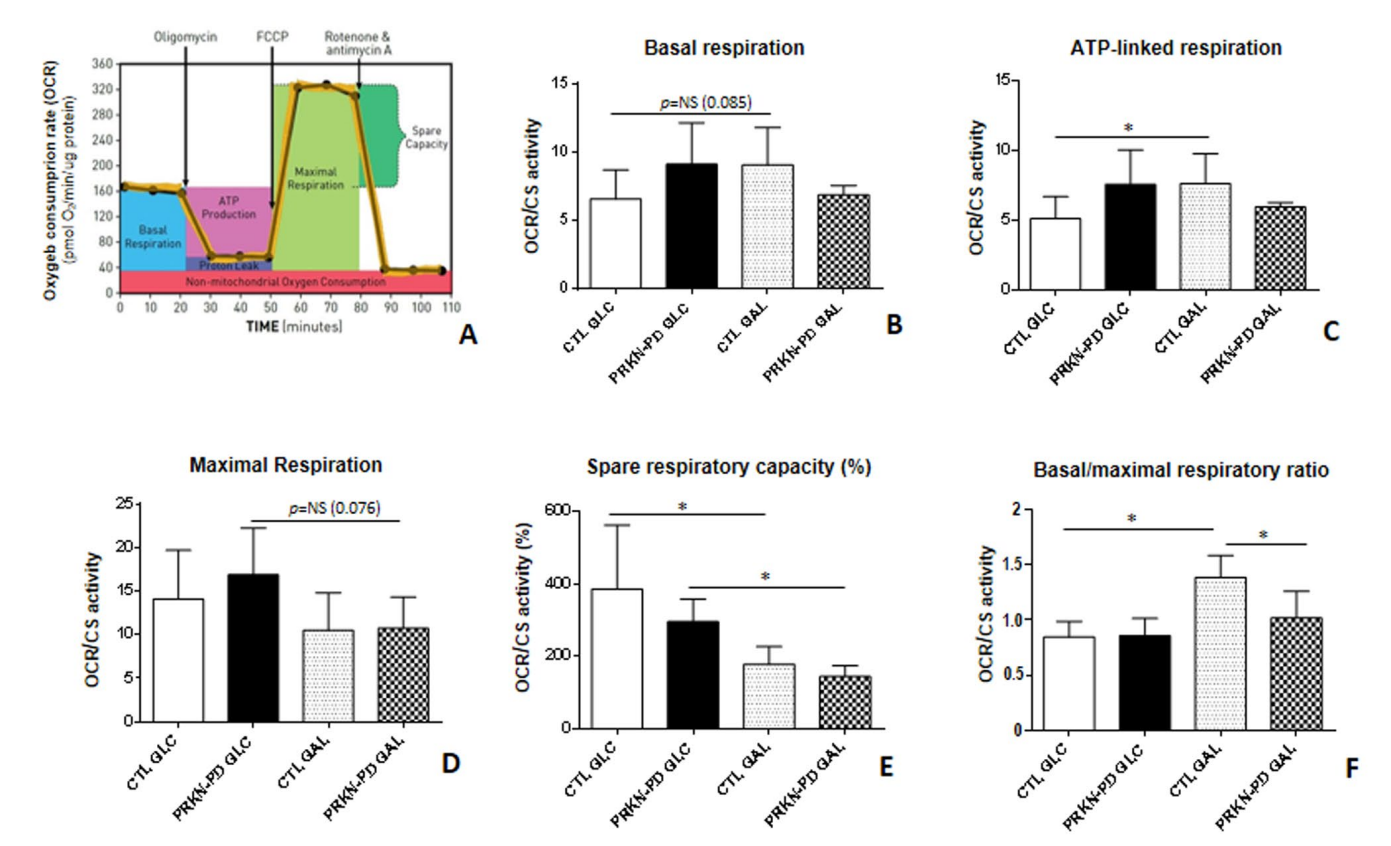
**Mitochondrial respiratory control ratios in control and PRKN-PD fibroblasts.** Illustrative mitochondrial respiration flux profile indicating respiratory control parameters (image obtained from Agilent Seahorse XF) **(A)**, basal respiration **(B)**, ATP-linked respiration **(C)**, maximal respiration **(D)**, spare respiratory capacity **(E)** and basal/maximal respiratory ratio **(F)**. In glucose, no significant differences were found between PRKN-PD and control fibroblasts in the respiratory control ratios although trends to increased basal and maximal as well as ATP-linked respirations and decreased spare respiratory capacity were observed. In galactose, PRKN-PD fibroblasts exhibited a significant decrease in basal/maximal respiratory ratio compared to the control fibroblasts as well as a downward trend in the basal respiration and ATP-linked respiration. Controls but not PRKN-PD significantly increased oxygen consumption linked to ATP production and the basal/maximal respiratory ratio in galactose compared to glucose. Both, control and PRKN-PD fibroblasts significantly decreased the spare respiratory capacity upon medium change. Each cell line was seeded in triplicate per condition (n=3 for GLC and n=3 for GAL). The results were expressed as means and standard error of the mean (SEM). *= p<0.05. CTL= Control fibroblasts. GAL= 10 mM galactose medium. GLC= 25 mM glucose medium. NS= not significant. OCR= Oxygen consumption rate. PRKN-PD= Parkin-associated PD fibroblasts. Respiratory control ratios were normalized by total protein content and by citrate synthase activity as a marker of mitochondrial content.

In order to assess the bioenergetic status of fibroblasts, we first performed high-resolution mitochondrial respiration analyses. The overall respiratory control ratios shown in Figure 1 B-F, are obtained from the respiration parameters illustrated in the mitochondrial respiratory flux profile (Figure 1A). In glucose, no significant differences in the respiratory control ratios were found between PRKN-PD and control fibroblasts. Even so, trends to increased basal, ATP-linked and maximal (uncoupled) respirations and a concomitant downward trend in the spare respiratory capacity were observed in PRKN-PD fibroblasts compared to controls. In galactose, PRKN-PD fibroblasts exhibited a significant decrease in basal/maximal respiratory ratio compared to the control fibroblasts. Although the rest of parameters were preserved, a downward trend in the basal respiration and ATP-linked oxygen consumption was shown. Upon changing from glycolytic to mitochondrial-challenging conditions, control but not PRKN-PD fibroblasts, significantly increased ATP-linked oxygen consumption and the basal/maximal respiratory ratio. In addition, both groups significantly decreased the spare respiratory capacity upon changing cells from glucose to galactose medium (Figure 1 A-E).

**Figure 2:**
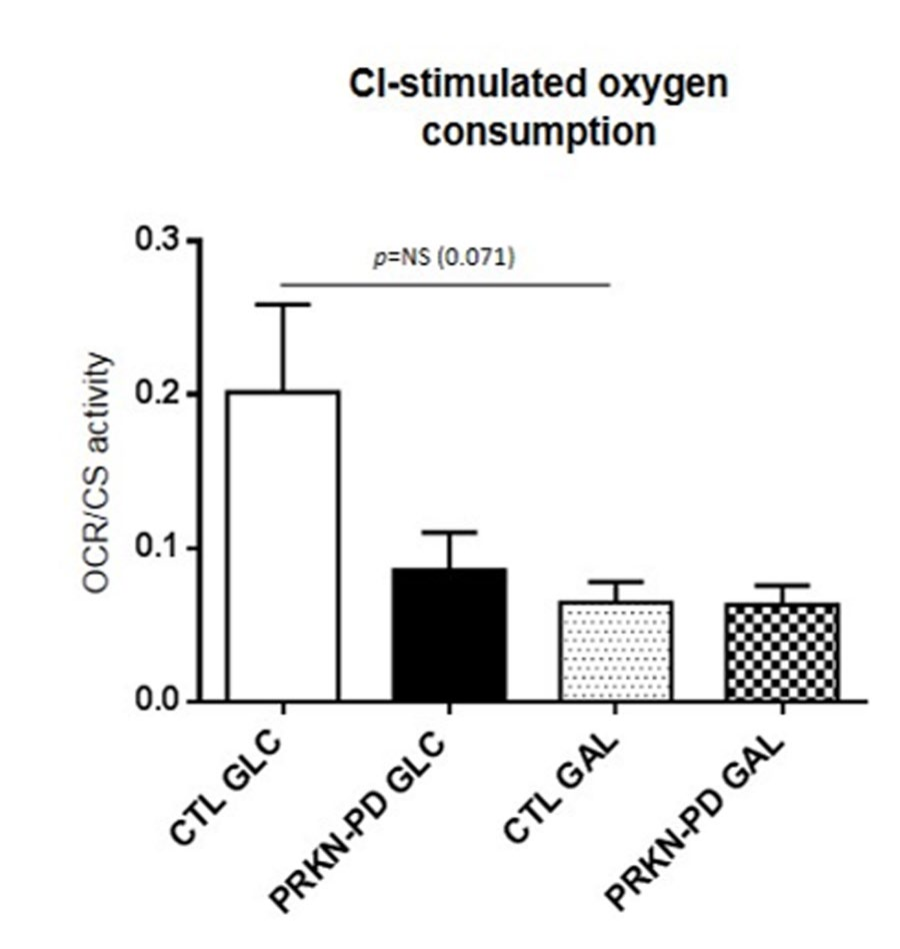
**Complex I-stimulated oxygen consumption through pyruvate and malate oxidation measurement** **in control and PRKN-PD fibroblasts**. No statistically significant differences were obtained between groups. In glucose, downward trends in CI-stimulated oxygen was shown in PRKN-PD compared to control fibroblasts. Exposure to galactose trended to reduce CI-stimulated oxygen consumption in control fibroblasts when compared to glucose, but not in PRKN-PD cells. The results are expressed as means and standard error of the mean (SEM). CTL= Control fibroblasts. GAL= 10 mM galactose medium. GLC= 25 mM glucose medium. NS= not significant. PRKN-PD= Parkin-associated PD fibroblasts. Oxygen consumption values were normalized by citrate synthase activity as a marker of mitochondrial content.

To understand the role of mitochondrial complex I (CI) in the overall oxygen consumption, which is reported to be affected in PD, we measured oxygen consumption through the specific oxidation of CI-substrates, pyruvate and malate (PMox). Although we did not obtain statistically significant differences, PRKN-PD showed a strong non-significant downward trend in CI-stimulated oxygen consumption compared to control fibroblasts in glucose. Exposure to galactose trended to reduce CI-stimulated oxygen consumption in control, but not in PRKN-PD fibroblasts, when compared to glucose (Figure 2).

### Mitochondrial respiratory chain enzymatic activities

**Figure 3:**
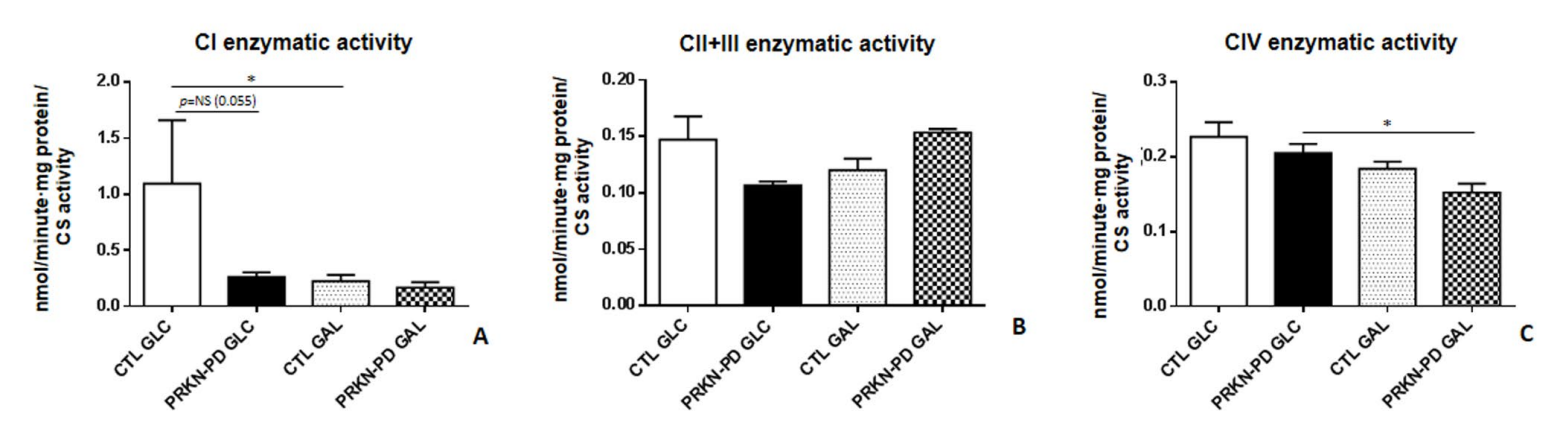
**Mitochondrial respiratory chain (MRC) enzymatic activities in control and PRKN-PD fibroblasts.** Enzymatic activities of the complexes I (**A**), II+III (**B**) and CIV (**C**) of the MRC. No significant differences were obtained between groups. Exposure to galactose significantly reduced CI-enzymatic activity of control fibroblasts and significantly decreased CIV enzymatic activity of PRKN-PD fibroblasts when compared to glucose. The results were expressed as means and standard error of the mean (SEM). *= p<0.05. CTL= Control fibroblasts. GAL= 10 mM galactose medium. GLC= 25 mM glucose medium. NS= not significant. PRKN-PD= Parkin-associated PD fibroblasts. Enzymatic activity values were normalized by citrate synthase activity as a marker of mitochondrial content.

We next wanted to determine if the differences observed in mitochondrial respiration analysis between PRKN-PD and control fibroblasts were reflected in the enzymatic activities of the Mitochondrial respiratory chain (MRC) complexes. Although no significant differences were obtained between groups, the same pattern exhibited in CI-stimulated oxygen consumption was reflected in CI enzymatic activity, where PRKN-PD showed a strong downward trend compared to control fibroblasts in glucose. Exposure to galactose significantly reduced CI-enzymatic activity of controls, but not PRKN-PD fibroblasts, when compared to glucose, in accordance to CI-stimulated oxygen consumption results. In addition, exposure to galactose significantly decreased CIV enzymatic activity in PRKN-PD fibroblasts when compared to glucose (Figure 3 A-C).

### Oxidative stress

**Figure 4:**
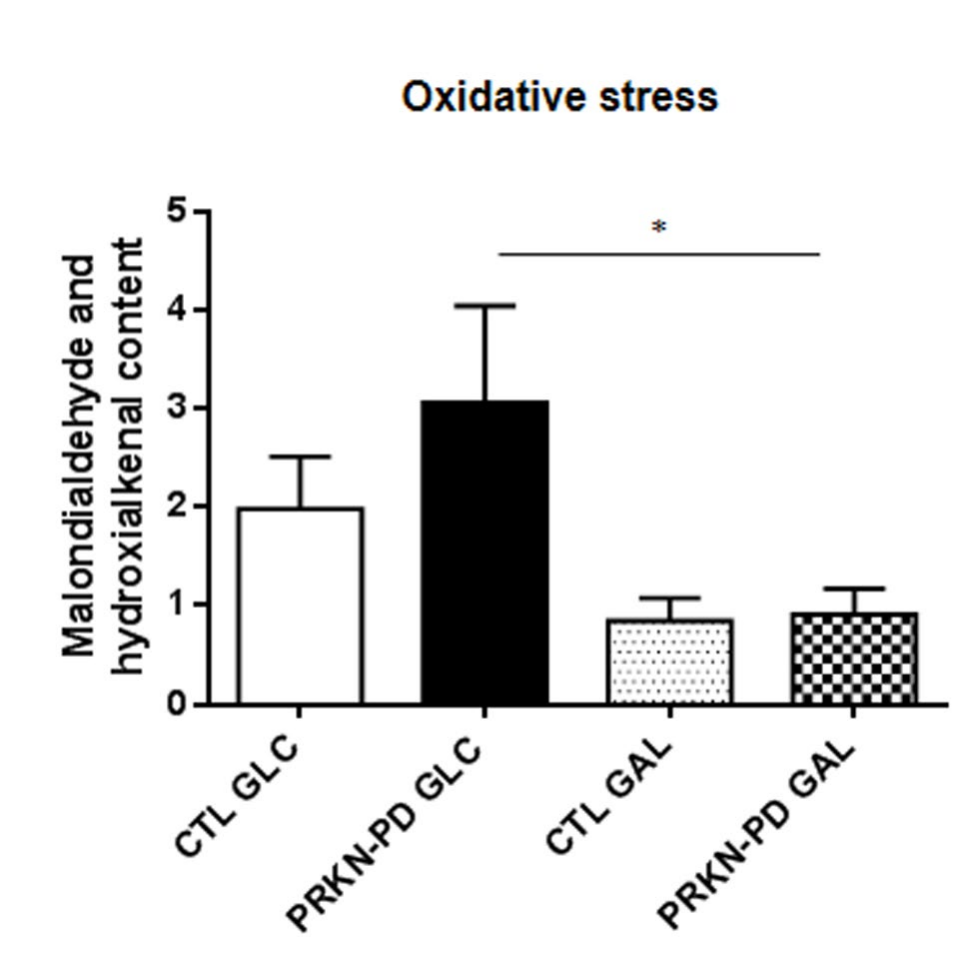
**Oxidative stress measured through lipid peroxidation in control and PRKN-PD fibroblasts.** In glucose, PRKN-PD exhibited an upward trend in lipid peroxidation compared to control fibroblasts while exposure to galactose significantly reduced oxidative stress levels in PRKN-PD compared to glucose. The results are expressed as means and standard error of the mean (SEM). *= p<0.05. CTL= Control fibroblasts. GAL= 10 mM galactose medium. GLC= 25 mM glucose medium. PRKN-PD= Parkin-associated PD fibroblasts.

Lipid peroxidation was measured as an indicator of ROS-derived oxidative damage. As illustrated in Figure 4, PRKN-PD exhibited an upward trend in lipid peroxidation compared to control fibroblasts in glucose, while exposure to galactose significantly reduced oxidative stress levels in PRKN-PD compared to glucose.

### Mitochondrial membrane potential

**Figure 5:**
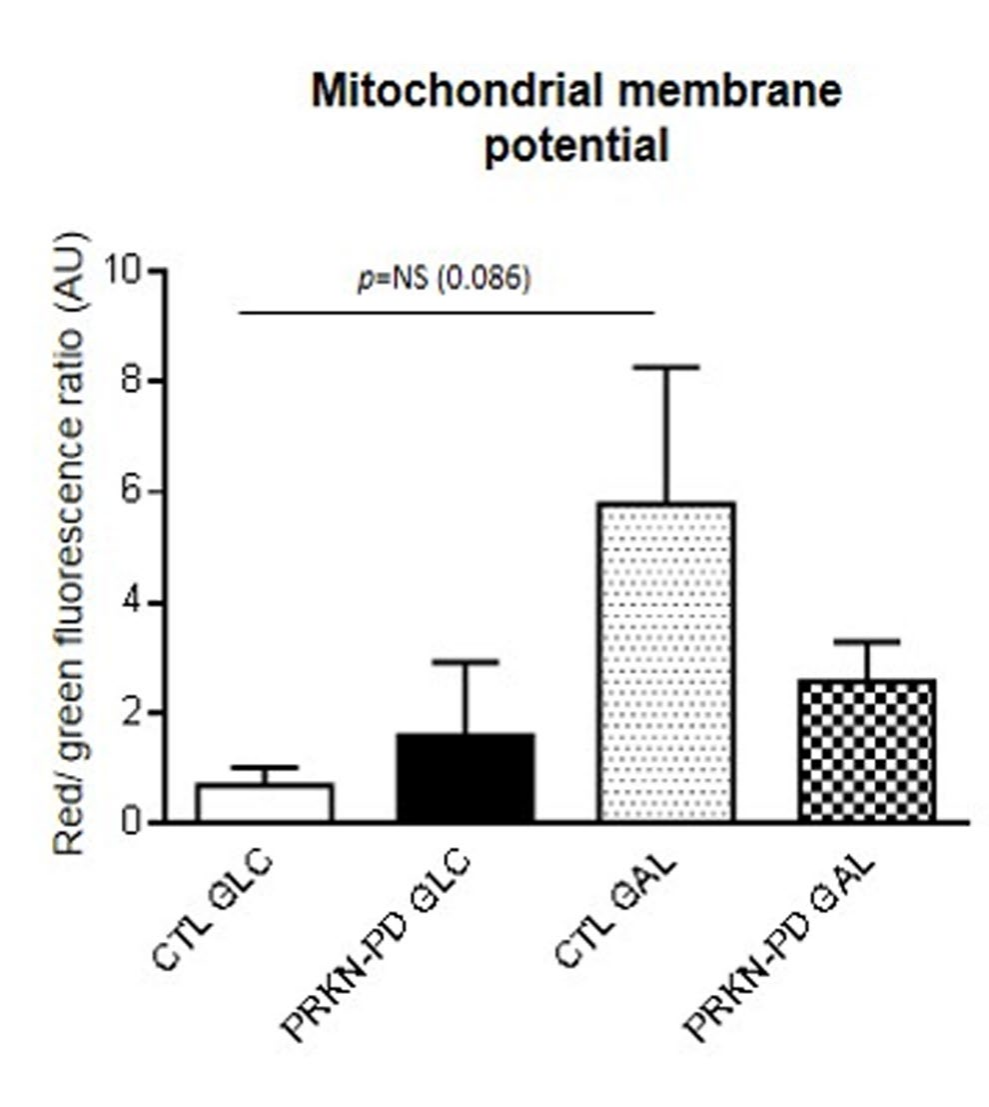
**Mitochondrial membrane potential in control and PRKN-PD fibroblasts.** Mitochondrial membrane potential is represented as the ratio of red vs. green fluorescence signals of JC-1 representing the cells with correctly polarized vs the cells with depolarized mitochondria. No significant differences in mitochondrial membrane potential were obtained between groups. Upon galactose exposure, control fibroblasts trended to enhance mitochondrial membrane potential as compared to glucose, while PRKN-PD fibroblasts remained unchanged. The results are expressed as means and standard error of the mean (SEM). AU= Arbitrary units. CTL= Control fibroblasts. GAL= 10 mM galactose medium. GLC= 25 mM glucose medium. JC-1: 5,5’,6,6’-tetrachloro-1,1’,3,3’-tetraethylbenzimidazol-carbocyanine iodide. NS= not significant. PRKN-PD= Parkin-associated PD fibroblasts.

Given that a correct mitochondrial polarization is crucial for mitochondrial integrity, we measured mitochondrial membrane potential in control and PRKN-PD fibroblasts. No significant differences in mitochondrial membrane potential were obtained between groups. However, upon galactose exposure, control fibroblasts trended to enhance mitochondrial membrane potential as compared to glucose, while PRKN-PD fibroblasts remained preserved (Figure 5).

### Mitochondrial network complexity

Mitochondrial function is a process intimately associated with changes in mitochondrial dynamics [37]. For this reason, we assessed mitochondrial length Aspect ratio (AR) and branching Form factor (FF) in control and PRKN-PD fibroblasts and found it was comparable between groups in both media (Supplementary Figure 1A and B).

### Mitochondrial content

**Figure 6:**
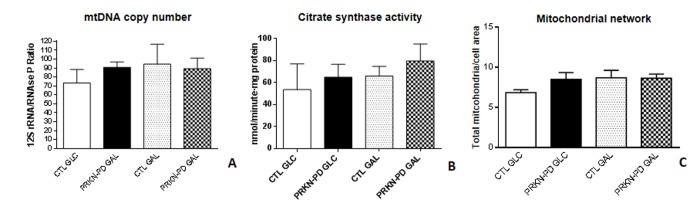
**Mitochondrial content in control and PRKN-PD fibroblasts**. Mitochondrial content was measured through mtDNA copy number (**A**), citrate synthase enzymatic activity (**B**) and mitochondrial network (**C**). Comparable mitochondrial content was observed between groups in both media. The results are expressed as means and standard error of the mean (SEM). CTL= Control fibroblasts. GAL= 10 mM galactose medium. GLC= 25 mM glucose medium. mtDNA= mitochondrial DNA. PRKN-PD= Parkin-associated PD fibroblasts.

We next evaluated mitochondrial content through the assessment of Mitochondrial DNA (mtDNA) copy number, Citrate synthase (CS) activity and mitochondrial network and found that all parameters were preserved between groups in both media (Figure 6 A-C).

### Autophagy

**Figure 7:**
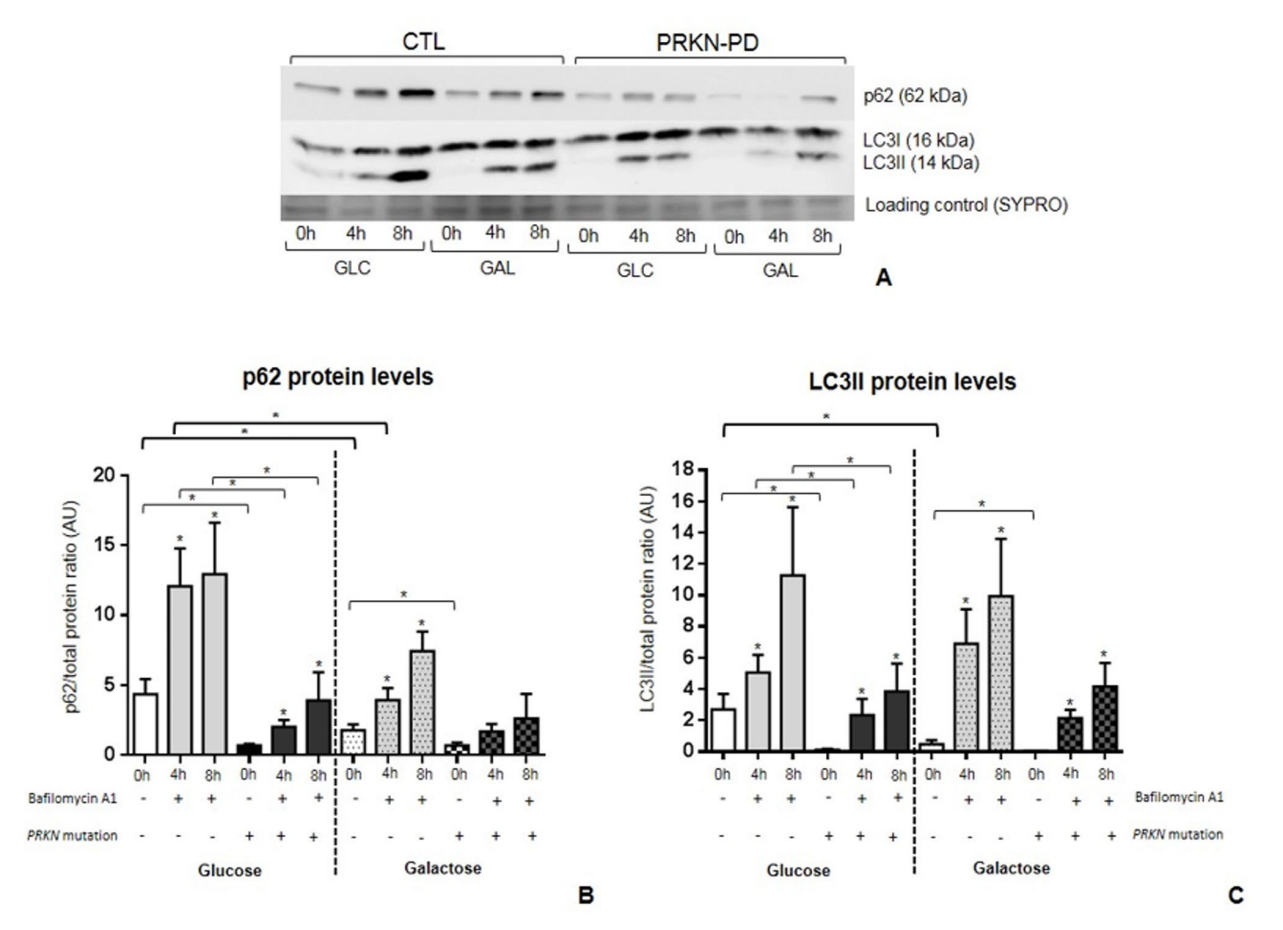
**Autophagic flux in** **control and PRKN-PD fibroblasts.** p62 (**A**) and LC3BII (**B**) protein levels at basal (0h) and under bafilomycin A1 treatment (4 or 8h) in glucose and galactose media. Basal levels p62 and LC3BII were significantly decreased in PRKN-PD compared to control fibroblasts in both media. PRKN-PD fibroblasts presented significantly lower p62 and LC3BII levels after 4 and 8 hours of treatment compared to controls in glucose and the same tendency was obtained in galactose. Exposure to galactose significantly decreased basal levels of both molecules compared to glucose in controls, but not in PRKN-PD fibroblasts. Controls, but not PRKN-PD, also showed significantly reduced p62 in front of conserved LC3BII protein levels upon treatment. Asterisks above the bars indicate statistically significant differences between protein levels at basal (0h) and after of bafilomycin A1 treatment (4 or 8h) within a group. The results are expressed as means and standard error of the mean (SEM). Asterisk brackets indicate statistically significant differences between CTL and PRKN-PD fibroblasts. Bold asterisk brackets indicate statistically significant differences between media. GAL= 10 mM galactose medium. GLC= 25 mM glucose medium. PRKN-PD= Parkin-associated PD fibroblasts.

To ascertain the potential impact of *PRKN* mutations on autophagy we quantified p62 and LC3BII protein levels. PRKN-PD fibroblasts presented significant lower basal levels of p62 and LC3BII in both media compared to controls as shown in Figure 7A and B (0h)**.**

The processes of autophagosome synthesis and degradation are spatially and temporally separated. In order to determine whether the decrease in basal p62 and LC3BII protein levels was due to a reduction in autophagosome synthesis or to a later stage in the pathway such as autophagosome degradation, we treated cells with bafilomycin A1. Bafilomycin A1 clamps fusion of autophagosomes with lysosomes and thus blocks autophagosome degradation, allowing measurement of autophagosome synthesis, whereas both synthesis and degradation occur in the untreated samples. The degradation rate can be deduced by subtracting LC3BII levels in the untreated samples from those treated with bafilomycin A1 [38].

**Figure 8:**
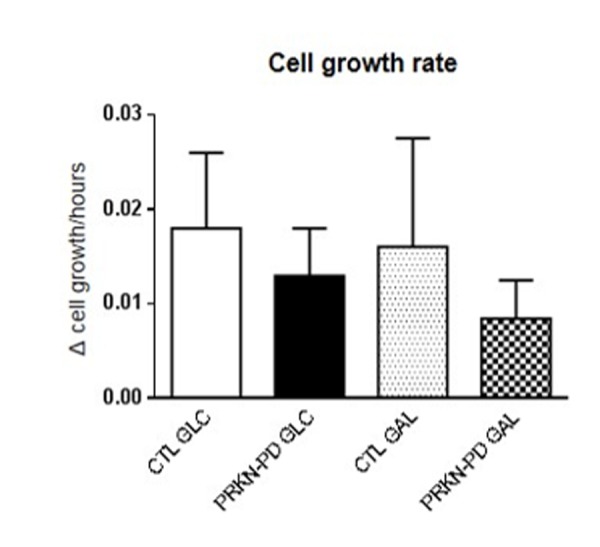
**Cell growth rate in control and PRKN-PD fibroblasts.** Although not significant, PRKN-PD fibroblasts showed downward trend in cell growth rate in both media. The results are expressed as means and standard error of the mean (SEM). AU= Arbitrary units. CTL= Control fibroblasts. GAL= 10 mM galactose medium. GLC= 25 mM glucose medium. PRKN-PD= Parkin-associated PD fibroblasts.

In both groups, p62 and LC3BII levels were significantly increased at 4 and 8 hours under bafilomycin A1 treatment compared to basal state, indicating some extent of autophagic flux in both media. However, in glucose, p62 and LC3BII levels at 4 and 8 hours of treatment were significantly lower in PRKN-PD compared to control fibroblasts and the same trends were observed in galactose (Figure 7 A and B; 4h and 8h). Upon exposure to galactose, controls but not PRKN-PD fibroblasts, showed significantly decreased basal levels of both molecules compared to glucose with concurrent significantly decreased p62 and conserved LC3BII protein levels upon bafilomycin A1 treatment, suggesting an enhancement of autophagic flux in these cells.

### Cell growth

We finally investigated cell growth rates in control and PRKN-PD fibroblasts in glucose and galactose media to assess how *PRKN* mutations impact on the overall cellular health. Although not significantly, PRKN-PD fibroblasts showed downward trend in cell growth rate in both media as shown in Figure 8.

## DISCUSSION

To our knowledge, this is the first study exploring the mitochondrial phenotype and the autophagic print in mitochondrial-challenging conditions (galactose) in skin-derived fibroblasts from PRKN-PD patients. Our results suggest that mitochondrial dysfunction and impaired autophagic flux are present in non-neural peripheral tissues of PRKN-PD patients. In addition, the alterations that PRKN-PD fibroblasts exhibited under mitochondrial-challenging conditions may be relevant to disease pathogenesis taking into consideration that the target tissue of the disease is predominantly oxidative.

To date, previous studies reporting altered mitochondrial homeostatic function in PRKN-PD fibroblasts have reported controversial results. Remarkably, all these studies were performed in glycolytic conditions that may partially unveil mitochondrial deficits [29–34]. In this sense, we first explored the mitochondrial function phenotype in glucose conditions and did not find any statistically significant difference between PRKN-PD and control fibroblasts. Interestingly, we found abnormal trend to increased overall mitochondrial respiration in PRKN-PD. More specifically, we observed a bias towards increased mitochondrial basal, ATP-linked and maximal respirations, as likewise reported by previous authors [31,32]. In contrast to these findings, a previous study that was performed with a different respirometry approach, described an overall decrease in all the aforementioned mitochondrial respiratory parameters of PRKN-PD fibroblasts [29].

To understand if the increased respiration in PRKN-PD cells was physiologically healthy, we assessed mitochondrial CI function as alterations at this level have been described in the CNS and in peripheral tissues of PD patients [33,39,40]. Although not statistically significant, we observed a strong downward decline in CI-stimulated oxygen consumption, which accordingly translated into a decreased CI enzymatic activity in PRKN-PD fibroblasts in glucose. In accordance with our results, Pacelli et al. and Mortiboys et al. [29,33] described CI enzymatic decline in PRKN-PD fibroblasts, while Grünewald et al. observed preserved CI function in isolated mitochondria from a larger cohort [34]. The association between the moderate increased mitochondrial respiration and the decreased MRC CI function in glucose is still a matter of debate but may correspond to an attempt of the PRKN-PD cell to overcome an inefficient oxidative phosphorylation (OXPHOS) function, mainly evidenced by CI deficiency.

To evaluate efficiency of the OXPHOS function, we measured oxidative damage which is a hallmark of mitochondrial dysfunction and has often been related with neurodegeneration and specifically with PD [6,41,42]. In accordance, we observed a non-significant strong upward trend in lipid peroxidation in the PRKN-PD group in glucose. This moderate increase in oxidative damage could be related to MRC malfunction in PRKN-PD fibroblasts suggested by both the abnormal increased mitochondrial respiration and CI dysfunction, considering CI a major source of ROS [43]. In line with our results, other authors demonstrated increased protein and lipid oxidation in smaller cohorts [29,34].

PRKN has been implicated in mitochondrial biogenesis [44]. In this context, we assessed mitochondrial content and observed it was preserved in PRKN-PD cells irrespective of the media, in accordance with Mortiboys et al. [33]. We cannot rule out that preserved PRKN-PD mitochondrial content may be triggered by an impairment of the mitochondrial turnover (mitophagy) in these cells that could counteract the lack of mitochondrial biogenesis. In contrast to our findings, Grunewald et al. reported increased mitochondrial content through an enhancement of the CS activity in isolated mitochondria of PRKN-PD fibroblasts as a compensatory effect for the mild mitochondrial dysfunction observed in these cells [34], whereas Pacelli et al. showed a decrease in this parameter [29]. Similarly, there is considerable controversy in regards of mitochondrial network morphology in PRKN-PD fibroblasts [29–34], although Parkin has been reported to be involved in mitochondrial dynamics [45]. In line with previous studies, we failed to demonstrate significant alterations in mitochondrial network morphology of PRKN-PD in neither of the media [30,31,34] despite the specific effectors of mitochondrial dynamics were not analyzed in the present work. Notwithstanding, a previous study reported decreased mitochondrial branching in PRKN-PD fibroblasts [32].

One of the main contributions of the present work is to evaluate the phenotype of PRKN-PD fibroblasts in mitochondrial-challenging conditions. Whereas the glycolytic metabolism of glucose forms pyruvate yielding 2 net ATP, the production of pyruvate via glycolytic metabolism of galactose yields no net ATP production, forcing the cells to rely on mitochondrial oxidative metabolism to obtain the vast majority of the cell ATP^28^ and approaching them somehow the neuronal metabolism. Moreover, replacing glucose with galactose medium has long been used to diagnose primary mitochondrial diseases in cells derived from patients with OXPHOS deficiencies [35,36]. This is the first study reporting that the exposition of PRKN-PD fibroblasts to oxidative conditions uncovers a differential mitochondrial function phenotype respect to standard glycolytic conditions. We specifically found a significant decrease in the mitochondrial basal/maximal respiratory ratio in galactose, which indicates that upon an increasing ATP demand that can only be confronted through enhancing mitochondrial metabolism, PRKN-PD cells cannot respond as controls do. Accordingly, we found downward trends in basal and ATP-linked respirations as well as in mitochondrial membrane potential in PRKN-PD compared to controls. In this line, Mortiboys et al*.* previously described decreased mitochondrial membrane potential in fibroblasts from five PRKN-PD patients in galactose [33]. Consistent with these results, we found a decline in mitochondrial CIV function and oxidative stress in PRKN-PD fibroblasts that was evident upon growing cells in galactose. Previous studies reported mitochondrial CIV deficiency in two PRKN-PD fibroblasts lines [29], while others have reported unaltered enzymatic activity of this complex in glucose medium [33,34].

The second main finding of this study is the description of autophagic alterations in PRKN-PD fibroblasts. Specifically, we observed significant lower levels of both the autophagy substrate (p62) and autophagosome content markers (LC3BII) in PRKN-PD fibroblasts at basal and after inhibiting autophagosome fusion with lysosomes in both media. These findings suggest that the overall autophagic flux is decreased in *PRKN* mutant fibroblasts. PRKN-PD fibroblasts did not exhibit changes in autophagic flux upon exposure to galactose as compared to glucose. Contrarily, our results suggest that galactose enhances autophagic flux in controls. Although p62 levels decreased in controls upon treatment when compared to glucose, p62 can be degraded through autophagosome-independent mechanisms such as the ubiquitin-proteasome system [46,47], and thus, may partially evade bafilomycin A1 treatment. The disruption in the autophagosome-lysosome pathway observed in PRKN-PD fibroblasts may promote accumulation of PRKN substrates including defective mitochondria and misfolded and aggregated proteins that, in physiologic conditions, should instead be degraded by the autophagic machinery. Thus, further research should be undertaken to investigate the role of autophagic disruption in PRKN-PD ethiopathogenesis.

In summary, exposure to galactose uncovered a differential mitochondrial phenotype in PRKN-PD fibroblasts compared to glycolytic medium while authopagic flux was reduced in these cells, irrespective of the culture medium. In addition, galactose suggested to enhance both mitochondrial function and autophagy in controls, by improving above all mitochondrial respiration performance and increasing autophagosome degradation. This improvement was not observed in PRKN-PD fibroblasts and may explain overall cell health decay evidenced in PRKN-fibroblasts by trends towards reduced cell growth. As neurons constitute a post-mitotic tissue that strongly relies on mitochondrial oxidative metabolism and autophagy to meet their high energy needs, alterations in these cell processes make them particularly vulnerable. Thus, the mitochondrial phenotype and the autophagic flux alterations exhibited by PRKN-PD fibroblasts under oxidative conditions may be of relevance to disease pathogenesis.

Notwithstanding, our study contains some limitations. First, despite being the study with a larger sample size respect to previous, sample size of our cohort is still limited. Second, the inherent individual variability present in the samples may also contribute to hinder inter-group statistical differences. Also, methodological issues amongst studies may support discrepancies. For instance, the use of different high-resolution respirometry approaches in which oxygen consumption is measured from seeding fibroblasts or from cells in suspension. Similarly, assessing MRC enzymatic activities in intact cells or in mitochondrial enriched fractions may contribute to outcome disparities. Third, although mitochondrial function has been previously reported as a critical regulator of autophagy in eukaryotic cells [48,49], the link between mitochondrial function and autophagy alterations in PRKN-PD fibroblasts is not herein explored. In this regard, future mechanistic studies in this field may be helpful in clarifying this issue. Finally, we acknowledge that many other mechanisms may be underlying the ethiopahtology of PD, where the loss of DAn may be the common eventual manifestation of this heterogeneous disease.

In conclusion, our work supports the view of PD as a systemic disease where mitochondrial and autophagic alterations play a role also in non-neural peripheral tissues such as skin fibroblasts, and encourages further studies in this model. The alterations that PRKN-PD fibroblasts exhibit under mitochondrial-challenging conditions may be relevant to disease pathogenesis and may be related to the increased susceptibility of patient DAn, which are predominantly oxidative, to undergo degeneration. Future studies with larger sample sizes and different PRKN-PD cohorts are warranted to confirm the results herein reported.

## MATERIALS AND METHODS

### Subjects and *PRKN* mutational screening

We recruited seven PD patients carrying mutations in the *PRKN* gene (PRKN-PD, n=7), and thirteen unrelated healthy subjects (CTL, n=13) among patients and relatives visiting the Movement Disorders outpatient clinic from the Hospital Clínic of Barcelona (Barcelona, Spain). PRKN-PD patients were diagnosed according to the UK Brain Bank criteria [50] and the mutational screening of the *PRKN* gene was performed as previously described [51]. Clinical and epidemiological data of PRKN-PD patients is summarized in Table 1. Gender and age-paired control group included six males and seven female subjects with an age range of 35-86 years and with a mean age of 58 years. Subjects with comorbidities, mitochondrial disorders, and those consuming mitochondrial toxic drugs were excluded from the study [52]. The study was approved by the ethics committee of the Hospital Clínic of Barcelona, following the guidelines of Helsinki declaration, and subjects were included in the study after signing the informed consent form.

**Table 1:** Clinical and epidemiological data of patients and control subjects providers of skin biopsy.

**Subject**	**Mutation**	**Gender**	**Age of disease onset**	**Age at skin punch biobsy**	**Treatment**
**PRKN-PD 1**	Heterozygous *PRKN* val15met	Male	47	57	L-Dopa
**PRKN-PD 2**	Homozygous *PRKN* exon 2-3-4 deletion	Male	35	69	L-Dopa
**PRKN-PD 3**	Homozygous *PRKN* exon 5-6 deletion	Female	27	63	L-Dopa
**PRKN-PD 4**	Compound heterozygous PACRG exon 1 deletion/ *PRKN* exon 6 deletion	Female	8	35	L-Dopa
**PRKN-PD 5**	Compound heterozygous *PRKN* exon 2 duplication/ exon 6 deletion	Male	20	49	L-Dopa
**PRKN-PD 6**	Homozygous *PRKN* exon 3 deletion	Male	25	48	L-Dopa
**PRKN-PD 7**	Compound heterozygous *PRKN* exon 6 deletion/ exon 8 deletion	Female	38	44	L-Dopa

No differences in mean age and gender were found between PRKN-PD and CTL subjects. Control group included six males and seven female subjects with an age range of 35-86 years and with a mean age of 58 years. CTL= healthy control subjects (not affected by PD or any *PRKN* mutation). *PACRG*=Parkin coregulated gene. PRKN-PD= Parkin-associated Parkinson’s disease. Val15Met= Methionine to Valine substitution at position 15.

### Cell culture

Fibroblasts were obtained by a 6 mm of diameter punch skin biopsy from the alar surface of the non-dominant arm of the subjects. Fibroblasts were cultured first in DMEM with high glucose (25 mM) supplemented with 10% (v/v) heat-inactivated fetal bovine serum and 1% (v/v) L-glutamine and penicillin/ streptomycin at 37ºC and 5% CO_2_ until 80-90% optimal confluence was reached. Each cell line was then exposed in parallel to 25 mM glucose (standard) or 10 mM galactose (mitochondrial-challenging) media for 24 hours [28,53–55]. Fibroblasts were harvested for further analyses as reported elsewhere [56]. All functional assays were performed in cells between passage 5 and 10.

### Mitochondrial respiration analysis

### *Mitochondrial oxygen consumption rates*

Oxygen consumption rates (OCRs) were measured in intact adherent fibroblasts through Agilent Seahorse XFe24 Analyzer (Seahorse Bioscience). Briefly, 35,000-40,000 fibroblasts per well were seeded in customized 24-well Seahorse cell culture plates and left to adhere overnight in 250 uL of growth medium. Each cell line was seeded in triplicate per condition (n=3 for glucose and n=3 for galactose). After 24 hours, growth medium was removed and wells were washed and replaced with Seahorse XF Base Medium (Seahorse Bioscience) containing either 25 mM glucose or 10 mM galactose plus 1 mM sodium pyruvate and 1 mM glutamine. Afterwards, plates were incubated for 30 min at 37 °C without CO_2_ according to manufacturer’s protocol. In order to determine the different respiratory control ratios, oxygen consumption was measured under basal conditions and after the addition of oligomycin, which blocks ATP synthase allowing the assessment of the natural proton leak across the inner mitochondrial membrane (IMM). This was followed by the addition of the uncoupler carbonyl cyanide-4-(trifluoromethoxy) phenylhydrazone (FCCP) to measure the maximal respiratory capacity. FCCP is an ionophore that directly transports protons across the IMM bypassing the ATP synthase proton channel thus leading to a rapid consumption of oxygen without the ATP generation. Finally, complex I and III inhibitors, rotenone and antimycin-A, were added to assess unspecific non-mitochondrial respiration (all reagents from Sigma-Aldrich). In addition, spare respiratory capacity and basal/maximal respiratory ratio were calculated as the maximal after basal OCR subtraction expressed in percentage, and the ratio between basal and maximal OCRs, respectively. All respiration values were normalized to total cell protein content determined in each well through the bicinchoninic acid assay (BCA) assay according to manufacturer’s protocol (Thermo Scientific) and CS activity (see mitochondrial respiratory chain enzymatic activities method section).

### *Mitochondrial complex I-stimulated oxygen consumption*

To assess oxygen consumption under the stimulation of mitochondrial CI, 1 million of fibroblasts were obtained and resuspended in ice-cold respiration MiR05 medium. High-resolution respirometry was performed in digitonin-permeabilized cells using Oroboros^TM^ Oxygraph-2k system® (Innsbruck, Austria) and oxidation of CI substrates, pyruvate and malate (PMox), was monitored following manufacturer’s protocol [56,57]. Results were normalized to cell number and CS activity (see mitochondrial respiratory chain enzymatic activities method section).

### Mitochondrial respiratory chain enzymatic activities

In order to study mitochondrial respiratory chain (MRC) function, enzymatic activities of mitochondrial complexes I (CI), II+III (CII+III) and IV (CIV) were spectrophotometrically measured at 37ºC in fibroblasts, as reported elsewhere [58,59]. CS activity was also spectrophotometrically determined at 37ºC in fibroblasts, as it is considered a reliable marker of mitochondrial content [60]. Mitochondrial content was further confirmed by alternative methods (see mitochondrial content method section).

All enzymatic assays were performed following national standardized methods and were run in parallel with internal quality controls [58]. Changes in absorbance were registered in a HITACHI U2900 spectrophotometer through the UV-Solution software v2.2 and were expressed as nanomoles of consumed substrate or generated product per minute and milligram of protein (nmol/minute·mg protein). All enzymatic activities were normalized by CS activity.

### Lipid peroxidation

Lipid peroxidation levels are indicative of the ROS-derived oxidative damage in cell lipid compounds. Lipid peroxidation was quantiﬁed using the BIOXYTECH® LPO-586™ colorimetric assay (Oxys International Inc., CA, USA). Specifically, the levels of malondialdehyde (MDA) and 4-hydroxyalkenal (HAE) which are peroxides derived from fatty acid oxidation, were quantified in duplicate through spectrophotometry. The results were next normalized by protein content and expressed as μM MDA+ HAE/mg protein, as previously reported [61].

### Mitochondrial membrane potential

Mitochondrial membrane potential of fibroblasts was assessed in a BD FACSCalibur^TM^ cell analyzer (BD Biosciences) by JC-1 potentiometric dye and both, green (~525 nm) and red (~590 nm) fluorescent emissions of each cell population were simultaneously monitored by flow cytometry, as reported elsewhere [62,63]. Results were obtained as percentage of cells with specific fluorescence indicating polarized or depolarized mitochondria. We calculated the red/green ratio indicating the ratio between correctly polarized and depolarized mitochondria, whose decrease indicates mitochondrial depolarization.

### Mitochondrial network complexity analysis

Mitochondrial content and mitochondrial network complexity were assessed by immunochemistry and confocal microscopy as reported elsewhere [56,64]. A minimum of 3 fibroblasts from each subject were visualized and analysed using the Image J software and a macro of instructions was used to perform semi-automatic quantitation [65]. Mitochondrial network of each cell was subjected to particle analysis and the following parameters were assessed: aspect ratio (AR) (major axis/minor axis), form factor (FF), which was calculated as the inverse of the circularity (4π·area/perimeter^2^) and, mitochondrial network or content (total number of mitochondria/total cell area). AR and FF values correspond to mitochondrial length and branching, respectively, and are considered parameters of mitochondrial health. AR and FF values of 1 are indicative of circular unbranched mitochondria which are a sign of pathologic mitochondrial isolation. As mitochondria elongate and become more branched, AR and FF values increase indicating mitochondrial health.

### Mitochondrial content

The following three approaches were used to quantify mitochondrial content in fibroblasts:

### *Mitochondrial DNA (mtDNA) copy number*

Total DNA from fibroblasts was isolated through the standard phenol–chloroform extraction procedure as previously reported [66]. To assess mitochondrial DNA (mtDNA) content, fragments of the highly conserved mitochondrial 12S rRNA gene and the constitutive nuclear ribonuclease P gene (RNase P) were amplified in triplicates by multiplex *q*RT-PCR using Applied Biosystems technology [67]. mtDNA content was expressed as the ratio between the 12S rRNA and RNase P genes.

### *Citrate synthase enzymatic activity*

CS activity was spectrophotometrically determined in fibroblasts (see mitochondrial respiratory chain enzymatic activities method section).

### *Determination of mitochondrial network*

Immunochemistry and confocal microscopy were performed to quantify mitochondrial network or content and expressed as the total number of mitochondria per total cell area (see mitochondrial network complexity analysis method section) [68]. In general, higher mitochondrial network values are considered sign of healthy mitochondria.

### Autophagic flux analysis

Where indicated, cells were treated with 0.1 µM bafilomycin A1 (Sigma) for 4 and 8 hours at 37°C in the presence of 5% CO_2_. Bafilomycin A1 is a proton pump inhibitor that neutralizes lysosomal pH preventing fusion of autophagosomes with lysosomes and thus allowing monitoring of autophagosome synthesis [38]. Briefly, fibroblasts were lysed with RIPA buffer (Sigma) plus protease inhibitor cocktail (Thermo scientific) followed by shaking and centrifugation at 16,100 g at 4°C for 10 minutes. Soluble fractions were kept at -80 °C until western blot analysis. Electrophoresis and blotting were performed, as reported elsewhere [56,62]. Blots were probed with anti-SQSTM1/p62 (Abcam) and anti-LC3B (Cell Signaling) antibodies. LC3BII is considered a marker of autophagosome number and p62 is an ubiquitin-binding scaffold protein that labels molecules and organelles that need to be degraded acting as a cargo receptor that is recruited to autophagosomes through LC3BII interaction [69]. Total protein content was obtained through SYPRO Ruby Protein Blot Stain, according to manufacturer’s protocol (Molecular Probes). The intensity of signals was quantified by densitometric analysis (Image Quant TL Software, GE Healthcare). Results were expressed as p62 and LC3BII protein levels normalized by the total cell protein content [70].

### Cell growth

Cell growth rate was manually determined through cell counting with the Neubauer chamber using trypan blue staining at the times of seeding and harvesting the cells [71]. Cell growth rate was calculated by applying the following formula:

**Figure:**
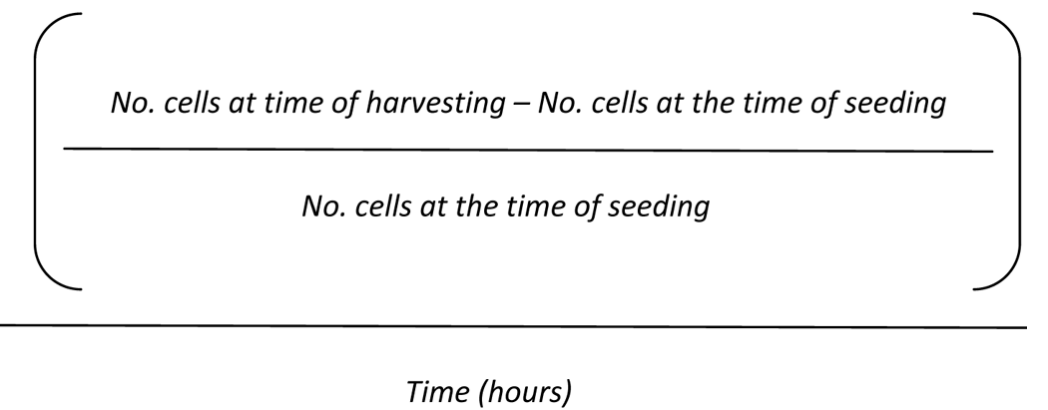
Formula.

### Statistical analysis

Statistical analysis was performed using the Statistical Package for the Social Sciences (SPSS, version 19) software (IBM SPSS Statistics; SPSS Inc). Differences amongst groups were sought by non-parametric tests after filtering for outlier values in the datasets. Specifically, Kruskal-Wallis and Mann–Whitney U statistical tests for independent samples were used when required. Significance was accepted for asymptotic 2-tailed p-values below 0.05 (for a confidence interval of α= 95%). Results were expressed as means ± the standard error of the mean (SEM).

## Supplementary Material

Supplementary Figure
