# Supplementary material for: Mitochondrial and autophagic alterations in skin fibroblasts from Parkinson disease patients with Parkin mutations
Source: Aging (Albany NY). 2019 Jun 9;11(11):3750–67. doi: 10.18632/aging.102014 (PMC6594812; doi:10.18632/aging.102014)
Supplement: Supplementary Figure [file aging-11-102014-s001.pdf]

SUPPLEMENTARY FIGURE

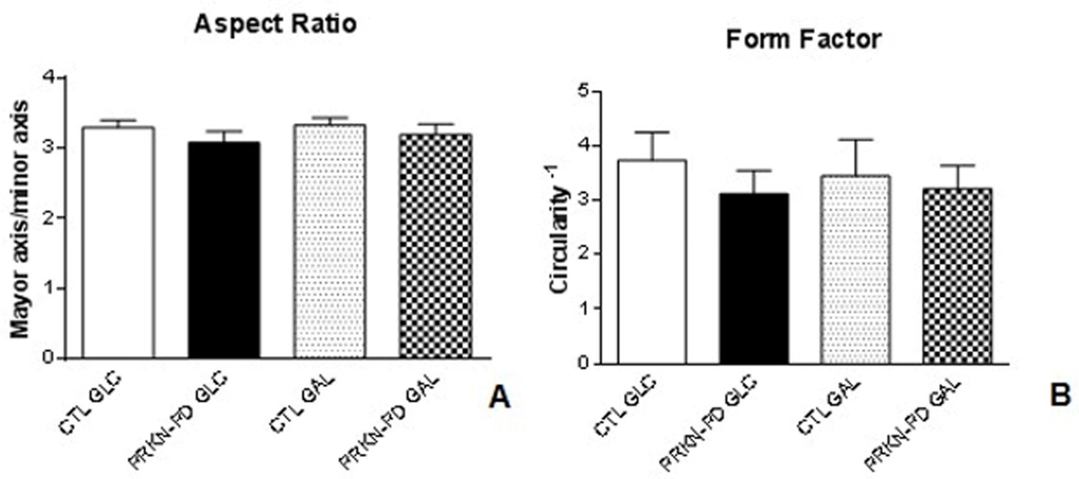

Supplementary Figure 1. Mitochondrial network complexity in control and PRKN-PD fibroblasts. Mitochondrial length (aspect ratio) (**A**) and mitochondrial branching (form factor) (**B**) were comparable between groups in both media. The results are expressed as means and standard error of the mean (SEM). GAL= 10 mM galactose medium. GLC= 25 mM glucose medium. PRKN-PD= Parkin-associated PD fibroblasts.
